# Cathepsin K Deficiency Impaired Ischemia-Induced Neovascularization in Aged Mice

**DOI:** 10.1155/2020/6938620

**Published:** 2020-06-30

**Authors:** Xueling Yue, Haiying Jiang, Ying Xu, Manli Xia, Xian-Wu Cheng

**Affiliations:** ^1^Department of Cardiology and Hypertension, Yanbian University Hospital, Yanji, Jilin 133000, China; ^2^Department of Physiology and Pathophysiology, Jiaxing University Medical College, Jiaxing 314001, China

## Abstract

**Background:**

Aging is a major risk factor for cardiovascular disease. Cysteine protease cathepsin K (CatK) has been implicated in the process of angiogenesis, but the exact roles of individual CatK in vessel formation during aging are poorly understood.

**Methods and Results:**

To study the putative role of CatK in ischemia-induced angiogenesis, we applied a hindlimb ischemia model to aged wild-type (CatK^+/+^) and CatK-deficient (CatK^−/−^) mice. A serial laser Doppler blood-flow analysis revealed that the recovery of the ischemic/normal blood-flow ratio in the aged CatK^−/−^mice was impaired throughout the follow-up period. On postoperative day 14, CatK deficiency had also impaired capillary formation. CatK deficiency reduced the levels of cleaved Notch1, phospho-Akt, and/or vascular endothelial growth factor (VEGF) proteins in the ischemic muscles and bone marrow-derived c-Kit^+^ cells. A flow cytometry analysis revealed that CatK deficiency reduced the numbers of endothelial progenitor cell (EPC)-like CD31^+^/c-Kit^+^ cells in the peripheral blood as well as the ischemic vasculature. In vitro experiments, CatK^−/−^ impaired bone-derived c-Kit^+^ cellular functions (migration, invasion, proliferation, and tubulogenesis) in aged mice. Our findings demonstrated that aging impaired the ischemia-induced angiogenesis associated with the reductions of the production and mobilization of CD31^+^/c-Kit^+^ cells in mice.

**Conclusions:**

These findings established that the impairment of ischemia-induced neovascularization in aged CatK^−/−^ mice is due, at least in part, to the reduction of EPC mobilization and the homing of the cells into vasculature that is associated with the impairment of Notch1 signaling activation at advanced ages.

## 1. Introduction

Aging is associated with a decreased ability to form new blood vessels in response to ischemia, and this is linked to higher rates of cardiovascular complications and diminished capacity for tissue regeneration. There is considerable interest in understanding the mechanisms of angiogenesis in humans at advanced ages. The process of new blood vessel formation is associated with extracellular matrix (ECM) remodeling, which involves various components of proteolytic systems such as the matrix metalloproteinases (MMPs) and serine proteases [1–4]. Several studies have shown that members of the cysteine protease cathepsin family also participate in the angiogenesis of pathophysiological conditions [5–8].

The Cat family member cathepsin K (CatK) is a mammalian cysteine peptidase that is sorted to endo-lysosomes and secreted into the extracellular space; it is also required for the degradation of type I collagen and elastin [9–14]. There is growing evidence of specific intra- and extracellular functions for Cats, and these functions have been shown to participate in cardiovascular pathogeneses [15–18]. Recent data revealed roles of CatK in pathological conditions such as renal disease, metabolic disorder, and atherosclerosis-based cardiovascular disease [18–20]. However, few studies have examined the roles of CatK in the angiogenesis of animals or subjects at advanced ages. Urbich et al. reported that CatL has a critical role in the integration of circulating endothelial progenitor cells (EPCs) into ischemic tissue and that CatL mediated angiogenesis; they also observed high expressions of CatK in EPCs [6, 21]. The results of clinical trials of stem cell and progenitor cell treatments for ischemic diseases in elderly subjects have been disappointing [22]. Samman Tahhan and colleagues recently reported a close relationship between circulating progenitor cells and clinical outcomes in elderly patients with acute coronary syndrome [23]. It is thus necessary to determine what improves the mobilization and function of EPCs in aging.

Notch signaling regulates embryonic patterning and binary cell fate decisions, and it plays critical roles in mammalian embryogenesis and angiogenesis in the vascular development of adult ischemic limbs [24, 25]. The ligand binding of Notch cleavage is followed by *γ*-secretase-mediated proteolysis within the transmembrane domain, where it interacts with RBP-J protein; the complex functions as a transcription factor for downstream target genes, which is essential for neurogenesis, myogenesis, hematopoiesis, and angiogenesis [26, 27]. Thus, the proteolysis strictly regulates the terminal cleavage event and Notch signal activity. However, as we previously reported, CatK-mediated Notch signaling activation is necessary for postnatal angiogenesis in response to ischemia in young mice [28]. Drachman proposed that these phenomena may also occur in elderly humans with multiple age-related changes and that they are precipitated by impaired microvascular function resulting primarily from decreased Notch-related angiogenesis [29].

We conducted the present study to determine the influence of the targeted deletion of CatK gene on ischemia-induced angiogenesis and vascularization in aged mice, and we attempted to clarify the mechanisms underlying the impaired neovascularization involving Notch signaling inactivation in mice at advanced age.

## 2. Materials and Methods

### 2.1. Animals

The investigation conformed to the Guide for the Care and Use of Laboratory Animals published by the U.S. National Institutes of Health (NIH Publication No. 85–23, revised 1996). The experimental protocol was approved by the Animal Studies Committee of Nagoya University. We generated CatK-deficient (CatK^−/−^) C57BL/6J mice by gene targeting in mouse embryonic stem cells as described [28], and the mice used in this study were ≧96 weeks old.

### 2.2. Mouse Model of Hindlimb Ischemia

CatK^−/−^ and wild-type (CatK^+/+^) littermate controls were subjected to unilateral hindlimb ischemic surgery under ketamine (70 mg/kg, Sankyo Pharmaceutical, Tokyo) and xylazine (4.6 mg/kg, Bayer, Pharmaceutical, Tokyo) anesthesia [30]. At the indicated time points after surgery, mice were euthanized by an overdose of a ketamine and xylazine mixture, and then skeletal muscles were dissected out and immediately frozen at liquid nitrogen temperature before they were stored at −80°C until the western blot analysis.

### 2.3. Ischemic Hindlimb Perfusion Assay

At the indicated time points, the ratio of blood flow in the ischemia to that in the normal limb was measured with laser speckle blood flow imaging (LSBFI, Omegazone, OZ-1, OmEGA Wave, Tokyo) as described in [30]. With LSBFI, the blood flow is shown as the changes in the laser frequency in pixels of different colors. Here, after the blood flow was scanned twice, the stored images were subjected to computer-assisted quantification, and the average flows of the ischemic and nonischemic limbs were calculated. The results of the quantitative analysis of blood flow are expressed as the ratio of left (ischemic) to right (nonischemic) LDBF to avoid data variations because of ambient light and temperature.

### 2.4. Measurement of Capillary Density

We assayed the capillary density in cross-sections of adductor muscle at postoperative day 14 with anti-CD31 monoclonal antibody (mAb) (Santa Cruz Biotechnology, Santa Cruz, CA). The endothelial cells were quantified by measuring the number of cells that were positive for CD31 per high-power field (400×) [30]. We measured five randomly chosen microscopic fields from 5 to 7 different sections in each tissue block to determine the number of capillaries per muscle fiber.

### 2.5. Western Blot Analysis

Proteins were isolated from muscle specimens by homogenization for 30 min in ice-cold lysis buffer (50 mM Tris-HCl, 1 mM EDTA, 150 mM NaCl, 1% Triton X-100, 0.25% SDS, pH 7.4)-supplemented protease inhibitor cocktail (1 tablet in 10 mL), 1 mM sodium orthovanadate, and 1 mM phenylmethylsulfonyl fluoride as described in [17]. Equal amounts (40 *μ*g) of proteins were subjected to sodium dodecyl sulfate polyacrylamide gel electrophoresis and electrophoretically transferred to a polyvinylidene difluoride membrane. The blots were incubated with primary antibodies: total Notch1, cleaved Notch1 (c-Notch1), total Akt, phosphor-Akt (p-Akt), and *β*-actin purchased from Cell Signaling Technology (Boston, MA). Vascular endothelial growth factor (VEGF) was purchased from Santa Cruz Biotechnology.

### 2.6. Immunocytofluorescence

For the immunofluorescence analysis, after their fixation and blocking, the corresponding sections of the muscle tissues at day 7 after surgery were incubated with fluorescein isothiocyanate-labeled CD31 monoclonal antibody and phycoerythrin-conjugated rat anti-mouse c-Kit (both BD Pharmingen, San Diego, CA) as described in [31]. Coverslips were treated with Prolong mounting medium (Molecular Probes, Eugene, OR) and visualized by confocal microscopy.

### 2.7. Analysis of EPCs

On ischemic postoperative day 7, peripheral blood (PB) and bone marrow were collected as described in [28]. EPC-like mononuclear cells (MNCs) in the PB and bone marrow were identified as CD31^+^ or c-Kit^+^ cells by flow cytometry. In brief, PB or bone marrow cells were pretreated with mouse anti-CD16^+^ and CD32^+^mAb to block the non-antigen-specific binding of immunoglobulins to the Fc*γ*III and Fc*γ*II receptors of monocytes. The PB or bone marrow cells were then incubated with a phycoerythrin-conjugated rat anti-mouse c-Kit mAb, followed by fluorescein isothiocyanate-conjugated rat anti-mouse CD31 mAb. Following treatment with flow cytometry lysing solution, the cells were centrifuged and suspended in phosphate-buffered saline for the flow cytometry analysis (total counted cells: 2 × 10^4^ MNCs). All antibodies for the flow cytometry analysis were from BD Pharmingen.

### 2.8. Bone Marrow-Derived EPC Isolation and Culture

Bone marrow-derived EPC isolation and culture bone marrow cells were isolated from aged CatK^−/−^ and CatK^+/+^ mice (*n* = 5 per group). After the isolation of mononuclear cells, bone marrow-derived c-Kit^+^ cells were isolated by using CD117 MicroBeads and magnetic-activated cell sorting (MACS) according to the manufacturer's instructions (Miltenyli Biotec, Bergisch-Gladbach, Germany). The c-Kit^+^ bone marrow cells were >90% positive for CD31^+^, as described [30]. The cultured cells were exhibited EC surface markers: CD31 and CD117. After being cultured on fibronectin-coated dishes in endothelial growth medium-2 (EGM-2: Lonza, Walkersville, MD) and 2% fetal bovine serum (FBS) for 7 days, the EPC-like c-Kit+ cells were collected and used for the cellular assay.

### 2.9. Cell Migration, Invasion, and Proliferation Assays

The cell proliferation assay was performed using the Cell Titer 96AO Assay kit (Promega, Madison, WI). Cells were seeded on gelatin-coated 96-well plates at 5 × 10^3^ cells in 100 *μ*L of endothelial basal medium-2 (Lonza, Walkersville, MD)/0.3% bovine serum albumin in the presence or absence of VEGF (50 ng/mL) for 48 hr and evaluated. The values of each group in triplicate were averaged and are presented as the absorbance's intensity.

Cell migration and invasion assays were performed using the Transwells of 24-well plates under hypoxic conditions. The cells that invaded and migrated to the outer side of the membranes were stained using Diff-Quik staining solution and calculated in 5–7 chosen fields of the triplicate chambers for each sample at high magnification (200x).

### 2.10. Tubulogenesis Assay

Cells at 2 × 10^4^ cells/well in a 24-well-plate were cultured for 24 hr on Matrigel in endothelial basal medium-2 containing 20 ng/mL of VEGF to induce a tubulogenic response under hypoxia conditions. Tubulogenesis was quantified using the BZ-II analyzer and Exe 1.42 software (Keyence, Osaka, Japan) to calculate the number and length of sprouts in six fields of each well.

### 2.11. Statistical Analysis

Data are expressed as means ± SEM. We performed a one-way analysis of variance (ANOVA) for comparisons of three or more groups, followed by Tukey's post hoc test or by Student's *t*-test (for comparisons of two groups) with SPSS software ver. 19.0 (SPSS, Chicago, IL). The blood flow data were subjected to a two-way repeated measures ANOVA and Bonferroni post hoc tests. Collateral capillary density was evaluated by two observers in a blind manner, and the values they obtained were averaged. Probability (*p*) values *p* < 0.05 were considered significant.

## 3. Results

### 3.1. Aging Impaired Angiogenesis in Response to Hypoxia

**Figure 1:**
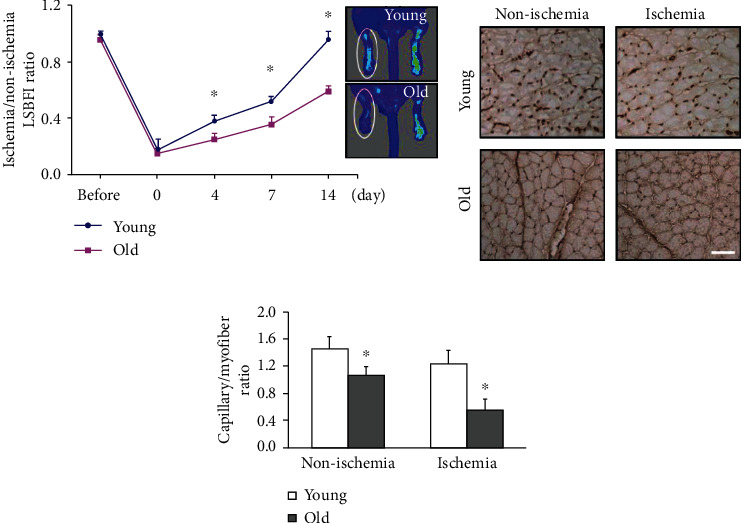
Aging resulted in impaired capillary formation and blood flow recovery in response to ischemia in the muscles of C57BL/6J mice. (a) Representative LSBFI result and quantitative data show the ratio of ischemia to nonischemia blood flow in young (8-week-old) and aged (96-week-old) CatK^+/+^ mice. (b) Representative images show the capillary density in nonischemic and ischemic thigh adductor muscles. (c) Quantitative analyses revealed that CatK^−/−^ impaired the capillary density in the ischemic muscle. Data are mean ± SEM (*n* = 6–8). ^∗^*p* < 0.05 vs. corresponding controls (days 4, 7, and 14) by two-way repeated-measures ANOVA and Bonferroni post hoc tests (i) or one-way ANOVA and Tukey's post hoc tests (ii).

**Figure 2:**
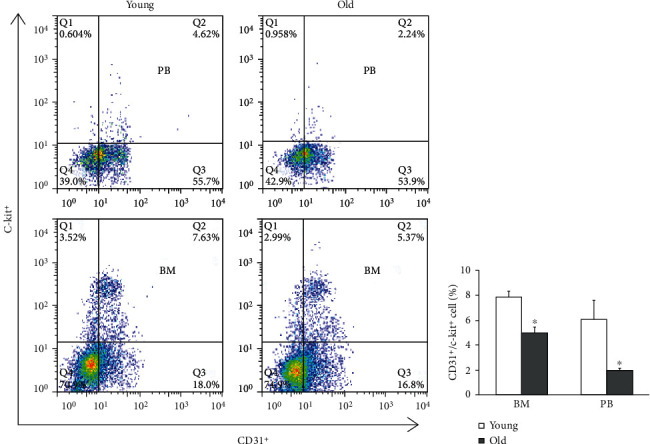
Aging impairs bone marrow endothelial progenitor cell (EPC) mobilization in CatK^+/+^ mice. (a, b) On operative day 7, representative flow cytometry plots and quantitative analysis results of the numbers of EPCs in bone marrow (BM) and peripheral blood (PB) of young and old CatK^+/+^ mice (counted total 2 × 10^4^ MNCs). Data are mean ± SEM (*n* = 6). ^∗^*p* < 0.01 by one-way ANOVA and Tukey's post hoc tests.

Our serial LSBFI analyses showed that the recovery of the ischemic/nonischemic blood flow ratio in the aged CatK^+/+^ mice remained impaired throughout the follow-up period (Figure 1(a)). On postoperative day 14, quantitative immunostaining revealed that the aged mice had lower capillary density in not only nonischemic but also ischemic muscles compared to the young mice (Figures 1(b) and 1(c)). The flow cytometry revealed that the numbers of EPC-like CD31^+^/c-Kit^+^ cells were lower in both the bone marrow and PB of aged CatK^+/+^ mice compared to those of the young control mice at day 7 after surgery (Figure 2). Aging therefore appears to impair ischemia-induced angiogenesis and vasculogenesis in mice.

### 3.2. CatK Deficiency Impaired Bone Marrow EPC-like CD31^+^/c-Kit^+^ Cell Mobilization

**Figure 3:**
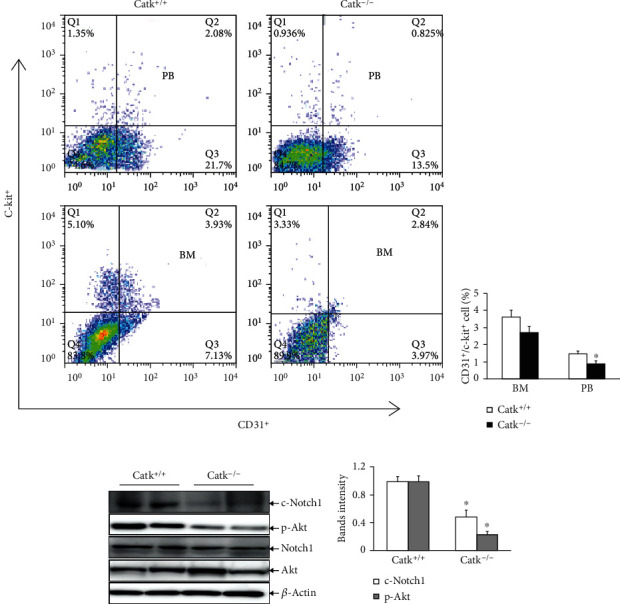
CatK^−/−^ impairs bone marrow-derived CD31^+^c-Kit^+^ EPC-like cell mobilization. (a, b) Representative flow cytometry plots and quantitative analysis data show the numbers of CD31^+^c-Kit^+^ cells in bone marrow (BM) and peripheral PB of both genotypes of old mice (counted total 2 × 10^4^ MNCs) at day 7 after ischemic surgery. (c, d) Representative immunoblot images and combined quantitative data show that CatK^−/−^ reduced the levels of c-Notch1 and p-Akt proteins in bone marrow-derived c-Kit^+^ cells. Data are mean ± SEM (*n* = 3–6). ^∗^*p* < 0.05 by ANOVA and Tukey's post hoc tests.

**Figure 4:**
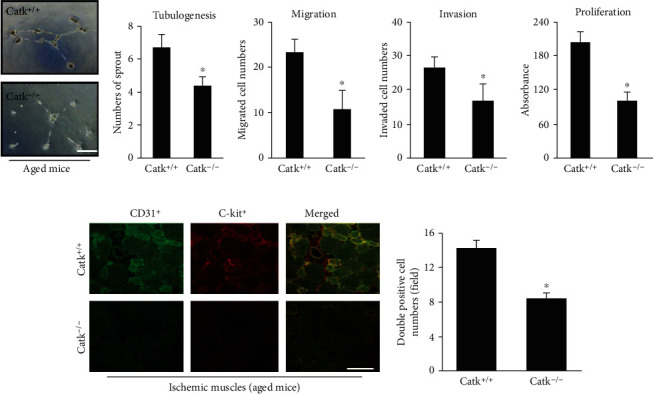
CatK deficiency reduced bone marrow-derived c-Kit^+^ cell angiogenic actions in aged mice. (a–e) c-Kit^+^ cells isolated by magnetic beads were applied to the tubulogenesis, migration, invasion, and proliferation assays. (f) Representative immunofluorescence and quantitative data show a reduction of CD31^+^c-Kit^+^ double-positive cells in the ischemic muscles of aged CatK^−/−^ mice compared to aged CatK^+/+^ mice. Scale bar: 50 *μ*m. ^∗^*p* < 0.01 by Tukey's post hoc test or Student's unpaired *t*-test.

Bone marrow-derived EPCs are known to play a partial role in postischemic neovascularization [3]. We determined the potential involvement of CatK in bone marrow EPC mobilization. We assessed the mobilization of EPCs from the bone marrow of CatK^+/+^ and CatK^–/–^ mice. The flow cytometry results demonstrated a marked reduction in the number of CD31^+^c-Kit^+^ cells in the PB of the CatK^−/−^ mice at day 7 after ischemia (Figures 3(a) and 3(b)). However, there was no significant difference between the genotypes in the number of EPC-like CD31^+^c-Kit^+^ cells in the bone marrow (Figures 3(a) and 3(b)). Simultaneously, Catk deficiency reduced the levels of c-Notch1 and p-Akt proteins in the bone marrow-derived c-Kit^+^ cells (Figures 3(c) and 3(d)). As shown in Figures 4(a)–4(e), CatK^–/–^ impaired bone marrow-derived c-Kit^+^ cell migration, invasion, proliferation, and tubulogenesis. On operative day 7, the immunocytofluorescence results also showed a dramatic reduction in the numbers of CD31^+^/c-Kit^+^ cells in the ischemic muscles of the aged CatK^−/−^ mice compared to the control mice (Figure 4(f)). These findings indicate that CatK deficiency may impair the mobilization of EPC-like cells from the bone marrow into the circulation to support the vasculogenesis that is associated with the reduction of c-Notch1/p-Akt signaling activation in the bone marrow EPC-like cells of aged mice.

### 3.3. CatK Deficiency Impairs Ischemia-Induced Angiogenesis

**Figure 5:**
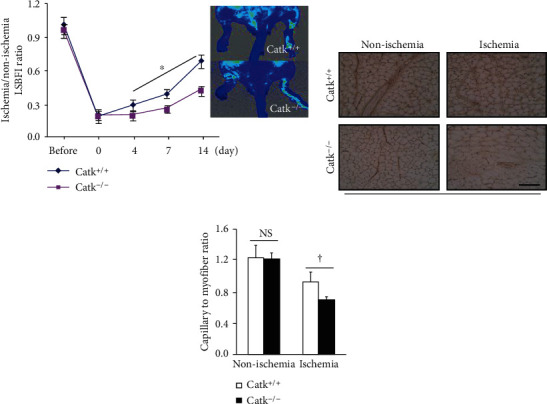
CatK deficiency impaired the blood flow recovery and capillary formation in ischemic muscles of aged mice. (a) Representative LSBFI results and quantitative data show the ratio of ischemia to nonischemia blood flow in the aged CatK^+/+^ and CatK^−/−^ mice. (b, c) Representative images and quantitative data show the capillary density in nonischemic and ischemic thigh adductor muscles. ^∗^*p* < 0.05, ^†^*p* < 0.01, vs. corresponding controls (days 4, 7, and 14) by two-way repeated-measures ANOVA and Bonferroni post hoc tests or one-way ANOVA and Tukey's post hoc tests. Scale bar, 50 *μ*m.

To determine whether a genetic ablation of CatK affects ischemia-induced angiogenesis in mice at advanced ages, we subjected CatK^−/−^ and CatK^+/+^ mice to hindlimb ischemic surgery. The serial LSBFI measurements showed that CatK^−/−^ inhibited the recovery of hindlimb perfusion throughout the follow-up period, and the ratio of ischemic to normal LSBFI was significantly lower in the old CatK^−/−^ mice compared to the age-matched control mice (Figure 5(a)). As anticipated, CatK deficiency persistently reduced the capillary density of the ischemic muscles (Figures 5(b) and 5(c)) Our findings thus indicate that CatK may contribute to impaired angiogenesis in aged mice in response to ischemia.

### 3.4. CatK Deficiency Reduced c-Notch1 in the Ischemic Tissues

**Figure 6:**
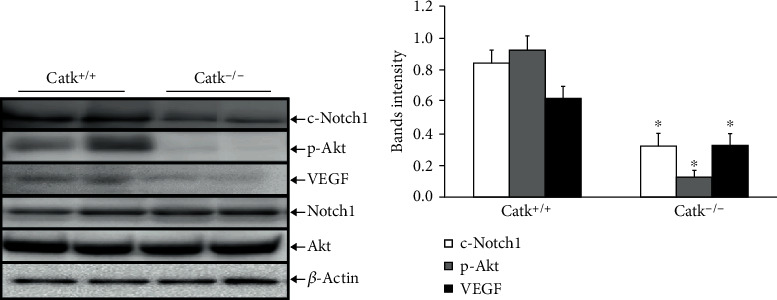
The levels of the targeted proteins in the ischemic muscles of aged CatK^+/+^ and CatK^–/–^ mice. (a, b) Representative immunoblot images and combined quantitative data show the levels of the c-Notch1, p-Akt, and VEGF proteins in the ischemic muscles of two groups. Results are mean ± SEM (*n* = 3). ^∗^*p* < 0.05 by one-way ANOVA and Tukey's post hoc tests.

As a potential mediator of angiogenesis, we assayed the expression of Notch1 protein in the ischemic tissues at day 7 after the operation. The results of the western blotting analysis demonstrated that CatK deficiency reduced the levels of c-Notch1, p-Akt, and VEGF in the ischemic tissues in the aged mice compared to the CatK^+/+^ mice (Figure 6). However, there was no significant difference in the Notch1 or Akt total protein expression between the two genotypes. There was also no significant difference in the levels of c-Notch1 in the nonischemic muscles between the CatK^+^/^+^ and CatK^−^/^−^ mice (data not shown).

## 4. Discussion

In aging, atherosclerosis-related critical ischemia and an attenuated ability to respond to ischemia are leading causes of limb amputation and poor cardiovascular disease prognosis. In this study, we evaluated the effects of CatK on aging-associated angiogenesis in mice at advanced ages, and our results indicate that CatK deficiency reduced the recovery of the blood flow and decreased the mobilization and homing of bone marrow-derived EPCs to support the ischemic vasculogenesis associated with the reduction in the Notch1 activation pathway.

The administration of bone marrow-derived or peripheral blood-derived EPCs has improved postischemic neovascularization in various experimental and clinical trials [22, 30, 32]. Impaired angiogenesis in individuals at advanced ages might be due to an interior decline in the regenerative capacity of vascular progenitors and/or a decline in a proregenerative niche. However, it has not been clear whether many proteinases are involved. Urbich et al. demonstrated that CatL and CatK were more strongly expressed by EPCs, and CatL was required for EPC-induced neovascularization in ischemic muscles [6]; they did not observe whether CatK affected the mobilization of bone marrow EPCs in aged animals. Our present flow cytometry and immunofluorescence analyses revealed that after ischemia, there were significantly fewer EPC-like CD31^+^/c-Kit^+^ cells in the PB and ischemic muscles of the aged CatK^−^/^−^ mice compared to the age-matched CatK^+^/^+^ mice. These data indicate that CatK has a critical role in the mobilization and homing of EPCs into ischemic vasculature in the neovascularization of aging. CatK has been shown to facilitate the angiogenic activity of EPCs in young mice, including invasion and tubulogenesis [28]. Pharmacological CatK inhibition exhibited an inhibitory effect on the proliferation ability of EPCs of young mice [28]. Our present findings demonstrated that aged CatK^−^/^−^ mice had lower levels of c-Notch1 and p-Akt proteins in their bone marrow-derived c-Kit^+^ cells compared to the control cells of aged CatK^+^/^+^ mice. Collectively, these observations suggest that Notch1/Akt signaling activation also acts as a key mediator of EPC function and mobilization in vascularization in mice under our experimental conditions.

Angiogenesis requires the degradation of the vascular basement membrane and remodeling of the extracellular matrix (ECM) in order to allow endothelial cells to migrate and invade into the surrounding tissues [1]. Many proteinases (i.e., MMP and serine protease family members) are involved in the degrading of ECM in angiogenesis processes [33]. In their review, however, van Hinsbergh et al. stated that other proteolytic pathways also play a role in angiogenic action in ischemic diseases [5]. Among the members of the cysteine cathepsin family, CatK is essential for extracellular type I collagen and elastin matrix metabolism [34], and it is a crucial candidate for matrix degradation during angiogenesis [28]. In the present study, we observed that CatK deletion impaired the blood flow recovery and capillary formation following hindlimb ischemia. We also observed that c-Notch1 and p-Akt were decreased by the ablation of CatK in aged mice. Notch1 receptor is a key molecule involved in angiogenesis in adults [26]. the Notch1 intracellular domain c-Notch1 is ultimately cleaved by the *γ*-secretase complex and translocates to the DNA-binding proteins to activate the transcription of downstream target genes of associated angiogenic factors [26]. Proteolytic activation is thus a very important step for Notch signaling transduction. We reported that CatK plays a role in the proteolytic processing of Notch1 with the sensitivity of the *γ*-secretase substrate to recombinant CatK [28]. The present results also indicate that in aged mice, CatK controls angiogenesis processes and that CatK is associated with Notch1 processing-related features via a VEGF/Akt signaling pathway. Takeshita et al. reported that endothelial Notch1 has a critical role in postnatal angiogenesis through an Akt pathway [25]. Collectively, these findings indicate that there is a relationship between Akt and Notch signal activation in angiogenesis regardless of age.

On the other hand, recent two comprehensive review articles substantiate the evidence for microvascular contributions to age-related vascular pathobiology that affect microvascular density, and also the recent findings showing the role of exercise in the reversal in microvascular rarefaction is potentially mediated by insulin-like growth factor-1 (IFG-1) [35, 36]. Accumulating clinical and laboratory evidence indicate the role of exercise-induced increases in blood flow in improving vascular integrity and stimulating neovascularization via the actions of IGF-1 in aged humans and animals. It is well-known that there is close-like between the degeneration and age-sensitive hormone IGF-1 levels in advanced age [37]. We previously reported that aging impaired neovascularization in response to ischemic stress, with decreased plasma VEGF levels that decline bone-marrow EPC mobilization and homing into the ischemic vasculature in animals [30, 31]. Thus, we speculated that the declined vascular regeneration ability might be at least partial, due to the impairment of EPC mobilization and functions, which was mediated by the age-sensitive hormone IGF-1 reduction in aged patients.

We reported the novel finding that CatK deficiency impairs the ischemia-induced neovascularization associated with the reduction of c-Notch-Akt signaling activation in bone marrow-derived EPCs and ischemic muscles of old mice. The identification of novel CatK targets designed to modulate vascular regenerative actions will contribute to therapeutic strategies to preempt limb amputation in elderly subjects.

## Data Availability

(1) All data used to support the findings of this study are included within the article. (2) All data used to support the findings of this study are available from the corresponding author upon request.

## Abbreviations

ANOVA: Analysis of variance
CatK: Cathepsin K
ECM: Extracellular matrix
EGM-2: Endothelial growth medium-2
LSBFI: Laser speckle blood flow imaging
MACS: Magnetic-activated cell sorting
MNCs: EPC-like mononuclear cells
MMPs: Matrix metalloproteinases
PB: Peripheral blood
SEM: Mean ± standard error of the mean
VEGF: Vascular endothelial growth factor.
